# Torpor use in response to predation risk in a small, free-living bird

**DOI:** 10.1093/beheco/araf069

**Published:** 2025-06-11

**Authors:** Alice Barratt, Justin Welbergen, Ben Moore, Christopher Turbill

**Affiliations:** Hawkesbury Institute for the Environment, Western Sydney University, Hawkesbury Campus, Science Rd, Richmond NSW 2753, Australia; Hawkesbury Institute for the Environment, Western Sydney University, Hawkesbury Campus, Science Rd, Richmond NSW 2753, Australia; Hawkesbury Institute for the Environment, Western Sydney University, Hawkesbury Campus, Science Rd, Richmond NSW 2753, Australia; School of Science, Western Sydney University, Hawkesbury campus, Richmond NSW 2753, Australia

**Keywords:** behavior, fear, foraging, heterothermy, superb fairy-wren, thermoregulation

## Abstract

Animal decisions trade-off the mortality risks of starvation and predation, and anti-predator behaviors generally incur a cost of reduced energy intake. Torpor and shallow rest-phase heterothermy are widespread physiological responses to starvation risk among small mammals and birds. Here, we present a field-based experimental test of the hypothesis that energy savings from torpor use can also reduce predation risk by moderating the energy cost of anti-predator behavioral responses in a small bird during winter. We manipulated perceived predation risk in wild populations of the superb fairy-wren (*Malurus cyaneus*) by playback of conspecific alarm calls during the daytime active-phase and tested for effects on body temperature measured continuously by telemetry during the nocturnal rest-phase. We found that alarm call playback was associated with subsequent rest-phase torpor bouts that were significantly deeper (minimum skin temperature: 28.7 ± 1.7 °C vs. 30.0 ± 1.5 °C) and longer (duration in torpor: 6.0 ± 2.7 h vs. 3.8 ± 2.3 h) compared to control periods. By demonstrating the connection between resting energy expenditure and energy costs of behavioral decisions during activity, our study has implications for understanding both the ecological functions of torpor and survival consequences of behavioral responses by small birds to environmental challenges.

## Teaser

Saving energy by cooling down while resting can offset reduced food intake when foraging under high predation risk. In a field experiment, we found that small birds respond to an increase in predation risk during activity by increasing their use of energy-saving torpor (a controlled reduction of metabolism and body temperature) while resting. Torpor use compensates for the energy cost of anti-predator behaviors, reducing mortality risk from both starvation and predation.

## Introduction

Animal survival depends on balancing a trade-off between the risks of starvation and predation (McNamara and Houston 1987). Foraging activity lowers starvation risk but increases exposure to predators, and predator avoidance behaviors often reduce energy intake. Prey can reduce their risk of predation by changes in behavior, such as reducing time spent foraging, selecting low-risk, low-yield foraging patches, and increasing rates of vigilance, but these responses often reduce foraging energy intake and so increase the risk of starvation (Verdolin 2006). Predation risk can incur further energy costs because of increased activity to evade predator attacks and increased resting energy costs of alertness and allostasis (Bobba-Alves et al. 2022). Engaging in anti-predator behaviors at the expense of resource acquisition can cause a reduction in body mass, growth rate, and reproductive output (Creel and Christianson 2008; Cresswell 2008) and hence reduce population growth rates (Hik 1995). Thus, physiological and behavioral mechanisms for reducing energy expenditure can increase survival when animals face environmental challenges because of consequences for both starvation and predation risk.

For small birds and mammals, the most effective physiological mechanism for reducing energy expenditure during resting is torpor. Torpor is a temporary and reversible state when metabolic rate and body temperature (T_b_) are reduced below normal levels (Geiser 2021). Traditionally, torpor use has been viewed simply as a response to an energy deficit caused by reduced food availability and/or high energy costs of thermoregulation in cold environments (Lyman et al. 1982). We now understand, however, that energy savings from torpor can have wide-ranging implications for many other aspects of an animal’s ecology (eg migration, reproduction, and survival; Geiser and Brigham 2012; Nowack et al. 2017). However, the energetic benefits of torpor must be weighed against the physiological and ecological costs associated with a reduced T_b_ and resting metabolism, including negative effects on cellular homeostasis, and reduced alertness and capacity to respond to a predator attack (Storey and Storey 1990; Humphries et al. 2003). In birds, reductions in T_b_ and metabolic rate are typically shallower than documented in mammalian torpor, and deep torpor (ie < 20 °C) appears to be restricted to the hummingbirds, swifts and nightjars (Apodiformes and Caprimulgiformes; McKechnie et al. 2023). Among the passerines that are known to use torpor, maximum reductions in T_b_ are often limited to about 10 °C (ie resting Tb of ~30 °C; Prinzinger et al. 1991; Reinertsen 1996; McKechnie et al. 2023). The lowest T_b_ so far recorded in a passerine bird is 22.9 °C (unpublished data in  Mckechnie et al. 2023). However, it is unclear if deep torpor in passerines is uncommon or reflects a lack of investigation. Recent studies using telemetry to study thermoregulation by free-living birds suggest that this form of shallow torpor might be more widespread among passerines than previously considered (Geiser 2019; Romano et al. 2019; Aharon-Rotman et al. 2021). Daily variation in torpor use is influenced within species by intrinsic factors, like body mass and energy need (Hiebert 1993), and environmental factors, such as food availability, thermal conditions and perceived predation risk (Laurila and Hohtola 2005; McKechnie et al. 2023).

An increased risk of predation while torpid is a key reason put forward for why passerine birds seem to avoid deep torpor (Pravosudov and Lucas 2000; McKechnie and Lovegrove 2002; Schleucher 2004; McKechnie et al. 2023). We could assume that torpor use increases immediate predation risk because torpid individuals are less responsive to stimuli and have decreased mobility (Rojas et al. 2012; Carr and Lima 2013; Boyles et al. 2020). However, it is also possible that torpor could make prey more difficult to find (Brown 1970) or reduce the incentive for a predator attack by remaining still (Hébert et al. 2019). Furthermore, it is not clear how torpor use during the rest-phase should relate to predation risk experienced during the active-phase. Only two studies have tested for an effect of predation risk on torpor use in birds, and both predicted that torpor use would be reduced when predation risk was higher. Domestic pigeons (*Columba livia*) when exposed to a flying model predator reduced T_b_ less during their rest-phase than without the model (Laurila and Hohtola 2005). In the only study investigating torpor and predation risk in a passerine bird, great tits (*Parus major*) when handled after sunset to simulate a predator attack reduced their T_b_ less during resting than without handling. However, a significant effect was limited to first-year birds and older great tits were not affected by simulated predation risk (Andreasson et al. 2019). One of the reasons for the mixed results could be a lack of clarity regarding whether perceived predation risk was increased during the active-phase or the rest-phase. This distinction is important because predation risk during the rest-phase could lead to reduced torpor use because animals are motivated to stay vigilant and responsive. In contrast, predation risk during the active-phase is likely to reduce energy intake, making an increase in rest-phase torpor beneficial.

Activity generally incurs a mortality cost, which is mostly because of increased predation risk, whereas resting is a much safer behavioral state (Lima and Dill 1990). The dormancy afforded by hibernation provides an exceptional case study among endothermic animals and monthly survival during hibernation is five-times higher on average for small mammals than during the active season (Turbill et al. 2011). Moreover, among populations of hibernating rodents, longer winter hibernation seasons in colder climates are associated with higher annual survival rates (Turbill and Prior 2016). Torpor use could be advantageous if the benefits of reduced exposure to active-phase predation that result from reduced energy needs outweigh the costs of any increase in rest-phase risk of predation. Experimental studies of mice and insectivorous marsupials in semi-natural environments provide support for this prediction, finding that an increase in perceived predation risk during activity resulted in a voluntary reduction in food intake and an increased use of rest-phase torpor (Turbill and Stojanovski 2018; Turbill et al. 2019). A benefit of torpor to survival because of a reduction in both starvation risk and active-phase predation risk has also been predicted by dynamic state-dependent modeling of optimal behavior during winter by small birds (Welton et al. 2002). However, to date there has been no experimental test of the hypothesis that foraging-associated predation risk is positively related to energy-saving torpor use in birds. Moreover, this hypothesis has not been tested in field experiments of wild animals in which perceived predation risk is manipulated against a background of natural environmental conditions.

We conducted a field-based experimental study to test the prediction that birds will respond to an increase in perceived predation risk during their active-phase with an increase in use of torpor during their rest-phase. As a model system, we focused on the superb fairy-wren (*Malurus cyaneus*), a small passerine (family Mauridae) known to use torpor (Romano et al. 2019). We increased the perceived risk of predation in wild-living superb fairy-wrens by exposing them to playback of conspecific aerial-type alarm calls combined with the display of model avian predators and used temperature telemetry to continuously record their T_b_ and hence quantify their response in depth and duration of rest-phase (nighttime) torpor use. Our experiment aims to determine if active-phase predation risk increases torpor use, thus indicating that small birds have a physiological capacity to reduce starvation risk and energy deficits caused by anti-predator behaviors. We aim to evaluate the interrelation between two independently well-studied but so-far poorly connected behavioral and physiological responses of small heterothermic endotherms: a reduction in foraging activity in response to perceived predation risk and an increase in torpor-facilitated energy savings during resting in response to reduced energy intake. Integrating these physiological and behavioral responses within a predation-starvation risk trade-off framework is important for understanding how small birds cope with environmental challenges.

## Methods

### Study species

The superb fairy-wren is a small passerine (8 to 10 g) that is common in south-eastern Australia. They are insectivorous ground-foraging birds that rest and forage in small social groups (Higgins et al. 2007). This species is a frequent model of behavioral ecology and it’s breeding system (Dunn and Cockburn 1999), vocalizations (Dalziell and Cockburn 2008), and antipredator behavior (Colombelli-Négrel and Evans 2017; McQueen et al. 2017) have been well studied. A recent study, conducted near our study site, found superb fairy-wrens routinely use torpor during winter, with an average nightly decrease in T_b_ (measured by skin temperature) of 14.7 °C (Romano et al. 2019).

### Study site and conditions

We conducted the study at two sites 650 m apart in the same continuous patch of woodland located on the Hawkesbury campus of Western Sydney University, which is on the western edge of Sydney, Australia (−33.62 °S, 150.73 °E). The study area consists predominantly of eucalyptus woodland, with some open grassland. During the study period, air temperature (T_a_), measured by temperature data loggers (HOBO MX2304, Onset Computer Corporation, Massachusetts, USA), ranged between an average daily minimum of 2.5 ± 3.1 °C (−2.0 to 8.8 °C) and a maximum of 17.2 ± 2.4 °C (10.8 to 20.6 °C). Weather conditions, sourced from a weather station (#067105) of the Australian Bureau of Meteorology located 5 km from the study site, documented that rainfall levels were low, totaling 9.6 mm over the study (Australian Bureau of Statistics 2023). The maximum daily wind speed averaged 32.2 ± 15.5 km/h and ranged from 11 to 63 km/h (Australian Bureau of Meteorology 2023).

### Experimental design

**Fig. 1.**
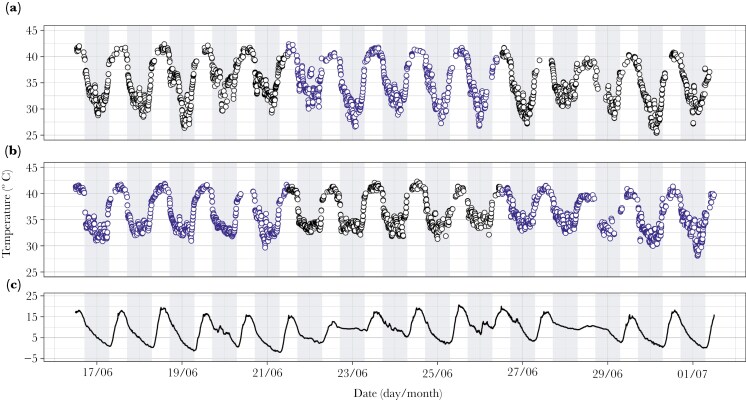
(*a* and *b*) Skin temperature of two example individuals of wild-living superb fairy-wrens and (*c*) air temperature recorded over the 15-d period of a field experiment conducted in winter using alarm call playback to increase the perceived risk of predation during foraging. Five-day periods of alarm call playback (blue circles) were alternated with undisturbed control periods (black circles), with an opposing sequence for birds in the two social groups (*a* and *b*). Grey vertical bars indicate times of night. Occasional gaps in data (more common during the daytime) indicate when birds foraged outside of the range of receiver dataloggers.

Our field experiment was designed to estimate the within-individual effects of changes in perceived predation risk on rest-phase torpor use in free-living superb fairy-wrens. We considered individuals at each of the two sites to be part of separate social groups and radio-tracking during the study supported this assumption because no individual moved between the sites. Birds were exposed to alternating 5-d periods of no disturbance (control) and an active-phase (daytime) sequence of alarm call playbacks (treatment) over 15 d in winter (16^th^ to 30^th^ June). Birds at each site experienced the 5-d control and treatment periods in opposing sequence and hence on different days (Fig. 1) to help overcome confounding effects of daily T_a_ and other weather variables (eg wind and rainfall) on torpor use (Geiser 2021). We continuously recorded the skin temperature (T_s_) of eight birds at each site (total 16 birds, female: 6, male: 10) over the duration of the study using temperature-sensitive transmitters.

### Treatment procedure

We increased the perceived risk of predation during the bird’s active-phase by broadcasting recordings of superb fairy-wren aerial alarm calls while simultaneously presenting a model of one of three locally common avian predators (grey butcherbird, *Cracticus torquatus*; pied currawong, *Strepera graculina*; or collared sparrowhawk, *Accipiter cirrocephalus*). Superb fairy-wrens give high-pitched aerial-type alarm calls (frequency range: 8 to 11 kHz) in response to a predatory bird in flight (Magrath et al. 2007; Fallow et al. 2013), and fly rapidly to cover and display higher vigilance when subjected to either the display of model birds of prey or the playback of aerial alarm calls (Magrath et al. 2007; Fallow and Magrath 2010; Fallow et al. 2013; McQueen et al. 2017). The same anti-predator behaviors are elicited in response to playback regardless of whether the aerial alarm calls were recorded in response to natural predators or in response to models (Leavesley and Magrath 2005).

We recorded alarm calls of superb fairy-wrens from other social groups at least 2 km distant from the study area on the Hawkesbury campus. We prompted alarm calls by ‘flying’ life-sized, paper-based, painted models of avian predators from a string on a pole near foraging superb fairy-wrens and recorded their acoustic responses using a microphone (Classic-Softie, Rycote Microphone Windshields Ltd., Leicestershire, United Kingdom) and digital recorder (R-26 6-Channel Portable Recorder, Roland Corporation, Shizuoka, Japan). In agreement with previous studies (Magrath et al. 2007), models of these species elicited aerial-type alarm calls. We visually identified alarm calls in Audacity ® 3.2.5 recording and editing software ( (Audacity (R)) based on frequency (range 8 to 11 kHz, peak frequency: 9.1 kHz) and presence of a single band that is rapidly frequency modulated about a constant carrier frequency (Fallow and Magrath 2010). Superb fairy-wren alarm calls consist of single or multiple individual call elements separated by intervals of less than 200 ms (Leavesley and Magrath 2005). An increasing number of call elements codes for greater urgency and prompts a stronger anti-predator response (eg faster fleeing to cover, longer latency to resume foraging; Leavesley and Magrath 2005; Fallow and Magrath 2010). We identified and selected 26 unique call elements for the playback sequences made for each study social group. Each call in our playback sequences contained 4 to 8 elements, indicative of ‘high-danger’ (McQueen et al. 2017). We kept the number of elements in each call consistent between study social groups. We composed 10 unique playback sequences for each social group by randomly arranging call elements. The total playback sequence was 10 min, of which 2.5 min consisted of call back and the rest was silence. Once composed, playback sequences were exported as uncompressed wave files.

The treatment was designed to artificially inflate the perceived presence of predators without interrupting the birds’ normal foraging for long periods. On every treatment day, three 10-min playback sequences were broadcast between 0700 and 1000 h, and between 1330 and 1630 h (total six sequences per day). There was a minimum of 15 min between the end of a playback and the start of another. A model predator bird was displayed when alarm calls were played to reinforce the playback; otherwise, it was concealed. The models (n = 3 different models) and playbacks (n = 10 sequences) were chosen randomly for each treatment cycle using a random number generator. All treatments were implemented by one person. To start a 10-min playback sequence, the researcher would radio-track an individual from the population to locate the foraging group. Tagged individuals typically foraged near each other, but once the group was sighted, we confirmed a minimum of four tagged individuals were present using the hand-held receiver. The playback was started within 4 to 7 m of the birds. Playbacks were broadcast using a single portable speaker (Roam, Sonos Inc., California, USA) worn across the researcher’s waist with the speaker facing forward. Playbacks were broadcast at a similar amplitude as natural alarm calls (Magrath et al. 2007), which was 60 dB when measured at a distance of 5 m (Sound level meter QM1591, Digitech Ltd., Victoria, Australia). Lastly, when the treatment sequence was completed, we left the study area and did not disturb the birds.

We avoided visiting the birds during control periods because we were likely to be perceived as a threat, especially after capture and the start of treatments. If it was necessary to check data loggers, one person visited the site, wore camouflaged clothes, worked quietly and only entered the site for the minimal amount of time (< 15 min).

### Recording skin temperature and air temperature

We caught superb fairy-wrens using mist nets (6 to 18 m long, 14 mm mesh size; 700 Series, Ecotone, Gdynia, Poland) set between 0630 and 1130 h and monitored at least every 5 min. Individuals were extracted, held in calico bags and processed on-site at a field table. We weighed individuals using a digital field balance (±0.1 g MS500, Pesola Ltd., Schindellegi, Switzerland), and assessed their plumage and coloring to determine sex and juvenile or adult (Menkhorst et al. 2017). Only adults were included in the study.

We attached pre-calibrated temperature-sensitive radio transmitters (0.33 g LB-2XT, Holohil Systems Ltd., Ontario, Canada) with a latex-based skin adhesive (SAUER skin adhesive 12% resin, Manfred-Sauer GmbH, Baden-Württemberg, Germany) to an unfeathered area between the feather tracts (ie an apterium) in the mid-dorsal interscapular region (Barratt and Turbill 2024). We radio-tracked the birds after sunset to determine the approximate night roost location and found that birds roosted in the same location for the duration of the study and subsequent tracking was not needed. We placed separate receiver/logger units near the roost location of each social group (R4500, Advanced Telemetry Systems Inc., Minnesota, USA) to record the pulse interval of each transmitter, and hence the T_s_ of birds, at 10 min intervals when they were near (within 200 m) the roost site. We checked the data loggers and downloaded the pulse interval data every 1 to 4 d. We calculated T_s_ from the recorded pulse interval (ms) using a 3^rd^-order polynomial equation fitted to calibration data (R^2^ > 0.99). Skin surface temperature is routinely used to infer T_b_ in small birds and mammals (McCafferty et al. 2015) and is assumed to be within 1°C of core T_b_ in small birds, particularly during resting when T_s_ is maximally insulated from T_a_ (Nord et al. 2013, 2016; Romano et al. 2019).

All procedures were approved by the Western Sydney University’s Animal Care and Ethics Committee (A14791) and a Scientific License (SL102713) under the New South Wales Biodiversity Conservation Act 2016.

### Data analysis

We excluded nights of T_b_ data from analyses when > 3 h of data were missing, which occurred on 27 bird-nights because of poor signal strength from some transmitters and the final nights when transmitter batteries began to fail. To reduce any effect of a carry-over in responses to perceived predation risk, we excluded data for the first 2 d of each 5-d control period that followed treatment. The final dataset used in analyses contained 178 nights of T_b_ data including 86 control nights and 92 treatment nights.

Data analysis was conducted using R Statistical Software version 4.3.2 (R Core Team 2021) as implemented by the software RStudio version 2023.09.0 + 463 (RStudio Team 2020). Figures were made using the package ‘ggplot2’ (Wickham 2016). We treated data between 1700 and 0800 h as the bird’s rest-phase and data from 0700 to 1700 h as the bird’s active-phase, aligning with sunrise and sunset times during the study. Median active-phase T_s_ was calculated between 0800 and 1700 h, only including days where > 20 data points were recorded. We calculated median active-phase T_s_ data over 141 bird-days, including 67 control and 74 treatment bird-days. We defined torpor using a simple and conservative threshold of 5 °C below resting normothermic T_s_ (Geiser 2021), which we estimated separately for each individual (Barclay et al. 2001) by averaging the lowest daily T_s_ recorded during the bird’s active-phase among control days (Aharon-Rotman et al. 2021). Torpor duration was calculated as the duration of time that T_s_ was below the individual torpor threshold.

Linear mixed-effects (LME) models were fitted by the restricted maximum likelihood method implemented by the “lmer” function in the ‘lme4’ package (Bates et al. 2015) to explain variation in torpor duration and depth (minimum nightly T_s_), and median T_s_ in the daytime. The package “bootpredictlme4” was used to generate confidence intervals using 1000 bootstraps (Duursma 2017), and model predicted effects were visualized using the package “visreg,” combined with plotting aesthetics from the package ‘ggplot2’ (Breheny and Burchett 2017). The global model fitted to explain median T_s_ during the daytime included maximum daily windspeed, body mass at capture, sex, and T_a_ averaged over the active-phase (from 0700 to 1700 h). Individual was included as a random effect on the intercept. The global models fitted to explain torpor bout duration and minimum nightly T_s_ both included maximum daily wind speed recorded during the preceding daytime foraging period (because of its possible influence on foraging success and other factors such as perceived predation risk), body mass at time of capture, sex, and average daily T_a_ (calculated from 0700 h of the preceding active-phase to 0700 h after the nocturnal rest-phase). Individual was included as a random effect on both the intercept and the slope when modeling torpor bout duration and minimum nightly T_s_ allowing both average levels and any effect of the playback treatment to vary among individuals. Rainfall was not included in any model because it only exceeded 1 mm per 24-h on 2 d during the study. Global models were simplified by sequential removal of least-significant terms.

## Results

Active-phase skin temperature varied among individuals and was not affected by the treatment. Daily minimum active-phase T_s_ (an index of individual resting normothermic T_s_) was 37.3 ± 1.2 °C (range: 35 to 39 °C). The average daily median active-phase T_s_ during the control period was 39.6 ± 0.6 °C (36.6 to 41.4 °C) and during the treatment period was 39.2 ± 0.7 °C (35.4 to 40.9 °C). The LME model fitted to explain variation in daily median active-phase T_s_ included only a significant positive effect of T_a_ and an effect of alarm call playback treatment was not significant (see Table 1 for statistics).

**Table 1.** Results of linear mixed-effects models fitted to explain variation in a) daily median active-phase (daytime) skin temperature (°C), b) minimum daily rest-phase (nighttime) skin temperature (°C), and c) torpor bout duration (h). N = number of individuals, n = number of observations.

a) daily median active-phase skin temperature (°C; marginal R^2^ = 0.065, conditional R^2^ = 0.587; N = 16, n = 204)					
fixed effects	coeff.	s.e.	d.f.	t-stat.	*p*-value
intercept	37.03	0.55	156	67.9	<0.001
air temperature (°C)	0.17	0.04	187	4.9	<0.001
alarm call treatment	−0.21	0.14	191	−1.5	0.16
random effects	**s.d.**				
individual identity (intercept)	0.98				

b) minimum daily rest-phase skin temperature (°C; marginal R2 = 0.07, conditional R2 = 0.46; N = 16, n = 178)					
fixed effects	coeff.	s.e.	d.f.	t-stat.	*p*-value
intercept	28.21	0.74	70	38.1	<0.001
air temperature (°C)	0.19	0.08	131	2.6	0.01
alarm call treatment	−1.10	0.31	11	−3.5	<0.001
random effects	**s.d.**				
individual identity (intercept)	1.28				
treatment | individual identity (slope)	0.57				

c) torpor bout duration (h; marginal R^2^ = 0.11, conditional R^2^ = 0.38; N = 16, n = 178)					
fixed effects	coeff.	s.e.	d.f.	t-stat.	*p*-value
intercept	7.11	1.07	89	6.7	<0.001
air temperature (°C)	−0.36	0.11	130	−3.2	0.002
alarm call treatment	1.96	0.44	13	4.5	<0.001
random effects	**s.d.**				
individual identity (intercept)	1.52				
treatment | individual identity (slope)	0.57				

**Fig. 2.**
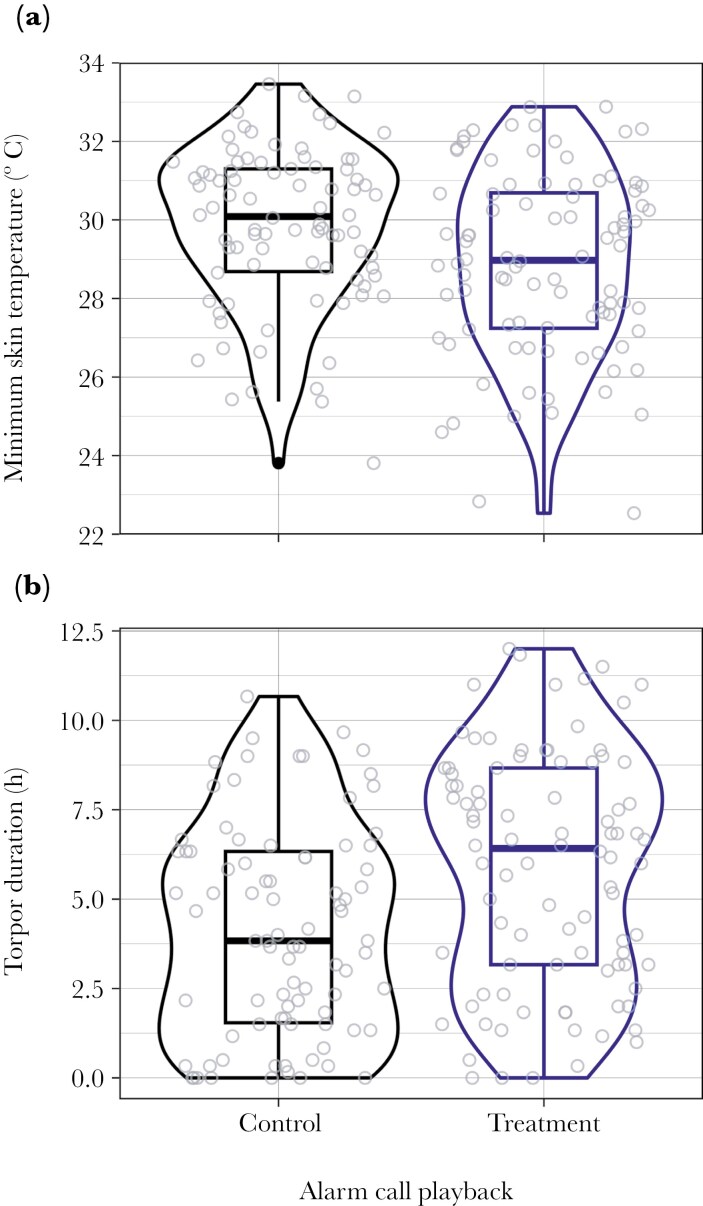
The distribution of (*a*) minimum nightly skin temperature (ie torpor depth) and (*b*) duration of torpor bouts exhibited by wild-living superb fairy-wrens during a field experiment using alarm call playback to increase the perceived risk of predation during foraging (number of birds = 16, number of bird nights = 178). The central box includes the median and defines the interquartile range, and minimum and maximum values are indicated by extending vertical lines. Raw data are represented by circles. Around each boxplot, lines illustrate the kernel probability density, truncated at minimum and maximum values.

**Fig. 3.**
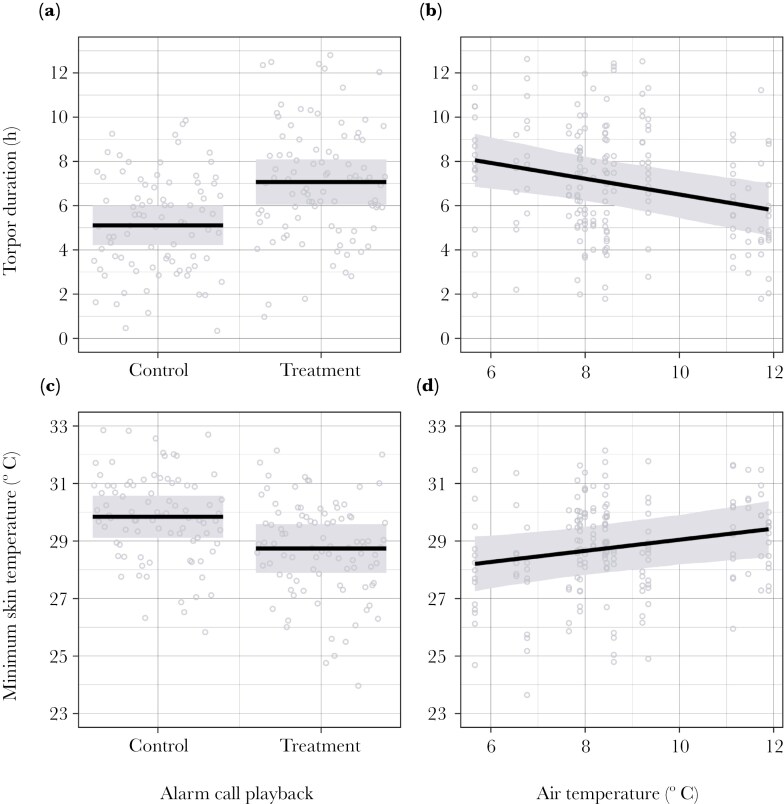
Model-predicted effects of (*a*) alarm call playback and (*b*) air temperature (°C) on torpor bout duration (h), and (*c*) alarm call playback and (*d*) air temperature (°C) on minimum skin temperature (°C) of wild-living superb fairy-wrens during a field experiment using alarm call playbacks to increase the perceived risk of predation during foraging. Shown are model-predicted significant mean effects (black lines), 95% confidence intervals (shading) and partial residuals (circles). See Table 1 for model test statistics.

The reduction in T_s_ during the nocturnal rest-phase was significantly deeper during the treatment compared to the control periods. The average daily minimum rest-phase T_s_ was 30.0 ± 1.5 °C (23.8 to 33.5 °C) during control periods and 28.7 ± 1.7 °C (22.5 to 32.9 °C) during the playback treatment periods (Fig. 2a). The LME model fitted to explain variation in minimum daily rest-phase T_s_ included significant effects of the alarm call playback treatment and T_a_. Minimum rest-phase T_s_ was 1.1 °C lower on average during the treatment compared to the control period (Fig. 3c), and higher with increasing T_a_ (Fig. 3d; Table 1).

The duration of torpor (ie time when T_s_ > 5 °C below individual normothermic T_s_) during the rest-phase was also significantly longer during the treatment compared to the control periods. The average daily torpor bout duration was 3.8 ± 2.3 h (0.0 to 10.7 h) during control periods, compared to 6.0 ± 2.7 h (0.0 to 12.0 h) during the playback treatment periods (Fig. 2b). The LME model fitted to explain variation in torpor bout duration included significant effects of the alarm call playback treatment and T_a_. Torpor duration increased by 2 h on average during the treatment (Fig. 3a) and decreased with T_a_ (Fig. 3b; Table 1).

## Discussion

We found clear evidence that a small passerine increased its torpor use in response to an elevated perceived risk of predation. On nights of treatment periods, following active-phase (day) exposure to alarm call playbacks, torpor bouts lasted around 2 h longer (an increase of 67%) and were also deeper (by 1.1° C), with the lowest T_s_ equal to the lowest minimum T_b_ known so far in any passerine bird (McKechnie et al. 2023). Treatment did not influence the T_s_ maintained during the active-phase. The energy savings gained by increased use of torpor during the rest-phase presumedly were required to compensate the daily energy budget for reductions in foraging energy intake during the active-phase caused by behavioral responses to the playback treatment. Our field experiment provides robust support for the prediction that small birds should increase torpor use during periods of increased foraging-associated predation risk because torpor provides the flexibility in daily energy budgets that is necessary for the use of anti-predator behaviors. Changes in thermoregulation are often studied in relation to environmental factors; however, biotic factors such as predation risk can also significantly influence rest-phase energy use (Morales et al. 2021). Moreover, rest-phase energy use is an important predictor of active-phase behavioral expression (Boratyński et al. 2024). These findings demonstrate the importance of integrating physiological mechanisms for energy savings with behaviors that affect foraging energy intake for understanding the consequences of combined responses to environmental challenges.

We found experimental evidence that energy savings gained by torpor are linked with changes in foraging behavior in response to artificially elevated perceived predation risk. Our treatment simulated the presence of predators through alarm call playbacks and the display of model predators, methods previously shown to prompt anti-predator behaviors in superb fairy-wrens (Magrath et al. 2007; Fallow et al. 2013; McQueen et al. 2017). We did not inhibit foraging directly by disturbance because for the vast majority of the day during the treatment birds were left undisturbed. Hence, it is highly likely the birds responded to the simulated predation risk with a voluntary reduction in foraging time or by foraging in safer but less productive ways, which incurred an energy cost that was accounted for by increasing torpor use. As a field experiment, we could not account for variation in the natural level of predation risk during the control periods and the magnitude of change in torpor use elicited by the treatment could have varied depending on background predation risk. Other treatment methods may also have induced a weaker or stronger “fear” response. The fact that torpor use was significantly increased, despite the relatively mild treatment and natural environmental conditions, provides robust evidence for a fundamental relationship between behavioral responses to predation risk during activity and use of energy-saving torpor use during resting.

In our experiment, the thermoregulatory response of superb fairy-wrens included a reduction in minimum T_b_ and an increase in the duration of the low T_b_ (ie torpor), which together would result in a reduction in resting energy expenditure compared to control days. It is noteworthy that, during the treatment period, we recorded a minimum T_b_ approximately equal to the lowest ever recorded in a passerine bird. Our measurement of a T_s_ of 22.5 °C is likely to indicate a similar core T_b_ to the minimum core T_b_ of 22.9 °C recorded for the southern double-collared sunbird (*Cinnyris chalybeus*) (unpublished data in McKechnie, 2023). Interestingly, minimum resting T_s_ was less affected by the treatment than torpor bout duration: whereas, on average, minimum T_b_ was reduced by 1.1 °C, torpor duration was increased by 2 h. This response could be interpreted as a strategy to increase energy savings while avoiding the costs associated with a reduction in T_b_ during torpor and could be important for birds which are restricted to shallow heterothermy. In accordance with our results, total rest-phase energy expenditure of hummingbirds using torpor is influenced more importantly by the duration of torpor bouts than by the energy savings achieved at lower T_b_ (Shankar et al. 2020).

Our study found a strong positive effect of active-phase predation risk on torpor use, but previous studies have found mixed effects of predation risk on torpor use. Differences in study outcomes could be due to the timing of perceived risk and the relative degree of predation risk during resting verse activity. For instance, one study on great tits found young birds, but not old, reduce their T_b_ less after a simulated predator attack via handling while in the nest box (Andreasson et al. 2019). Whereas another study found when a model predator was displayed outside the nest box (ie the threat was related to leaving the nest box and being active) great tits increased sleep, which is similarly expected to represent a trade-off between energy conservation and impaired awareness of predators (Stuber et al. 2014). Further, roosting behaviors that could provide protection against predators, like the use of hollows, might reduce the influence of predation risk during resting on torpor use. For example, great tits did not change their torpor use in response to chemical cues of predation introduced while birds were roosting in nesting boxes (Amo et al. 2011) but domestic pigeons, which did not roost in a hollow, attenuated torpor use in response to predation risk (Laurila and Hohtola 2005). We agree with the outcomes of models predicting optimal behavior for survival during winter by small birds (Pravosudov and Lucas 2000) that expectations for use of torpor as a function of predation risk will be strongly dependent on the relative degree of predation risk experienced during the active- versus the rest-phase.

A positive effect of torpor on survival because of reduction in both starvation risk and foraging-associated predation risk is likely a widespread mechanism in mammals and birds. Firstly, torpor and shallow heterothermy is a widespread response to decreased energy intake (Geiser 2021; McKechnie et al. 2023). Among animals that do not use torpor, minor reductions in rest-phase T_b_ can be invoked in response to a critical energy deficit and result in substantial energy savings (Ben-Hamo et al. 2010; Hohtola 2012). Further, for birds that exhibit only shallow heterothermy that does not meet common definitions of torpor, reductions in resting T_b_ are responsive to environmental conditions (Dolby et al. 2004; Nord et al. 2011; Barratt and Turbill 2024), suggesting shallow heterothermy is plastic and can be used as a facultative response to energy loss. Secondly, our finding is congruent with outcomes from dynamic state-dependent modeling, which predicted birds select lower-yielding but safer foraging patches when the risk of starvation overnight is reduced by small increases in rest-phase heterothermy (Welton et al. 2002). Experiments manipulating perceived predation risk for small mammals under semi-natural conditions also found a reduction in foraging energy intake and a compensatory increase in use of torpor during resting (Turbill and Stojanovski 2018; Turbill et al. 2019). And despite increasing avoidance behaviors in response to predation risk, body mass in small mammals was not affected, suggesting heterothermy sufficiently offset the loss in foraging intake associated with anti-predator behaviors (Morales et al. 2021). Finally, using torpor to reduce predation risk, as well as starvation risk, appears to have positive outcomes on survival. For example, torpor use was predicted to increase overwinter survival rates of small birds by lowering mortality rates from both predation and starvation (Welton et al. 2002). Further, comparative data on survival in wild mammals suggest that the energy savings from torpor can reduce foraging requirements, thereby minimizing exposure to predators and contributing to lower extinction rates in heterothermic mammals (Geiser and Turbill 2009; Liow et al. 2009; Hanna and Cardillo 2014). Accumulating theoretical, comparative, and experimental studies are finding support for the hypothesis that torpor enhances survival not only by reducing immediate starvation but also by reducing predation risk associated with foraging activity. Hence, energy savings from torpor and shallow rest-phase heterothermy should be integrated in our understanding of behavioral responses to environmental conditions and resulting outcomes on survival.

In conclusion, our field-based experiment found that the simulation of increased predation risk during the active-phase caused an increase in the depth and duration of rest-phase torpor use in a small passerine bird. We hypothesize that the rest-phase energy savings gained by torpor allow for a greater investment in anti-predator changes in foraging behavior. Our findings support expectations of theoretical models that torpor use increases survival for a small bird in winter because torpor energy savings can help resolve the trade-off between starvation and predation risks. We suggest these findings are generally applicable among endotherms because rest-phase hypothermia and torpor are common mechanisms for energy conservation (Geiser 2021; McKechnie et al. 2023) and broadly similar results have been found in previous experimental and comparative studies for heterothermic mammals (Liow et al. 2009; Turbill and Stojanovski 2018; Turbill et al. 2019). Consequently, rest-phase energetics appear to be a fundamental factor in active-phase behavioral decisions for resolving the trade-off between the risks of starvation and predation.

## Data Availability

Analyses reported in this article can be reproduced using the data provided by Barratt et al. (2025).
